# Supplementary material for: Examining the Use of Temporal-Difference Incremental Delta-Bar-Delta for Real-World Predictive Knowledge Architectures
Source: Front Robot AI. 2020 Mar 13;7:34. doi: 10.3389/frobt.2020.00034 (PMC7805647; doi:10.3389/frobt.2020.00034)
Supplement: Supplementary file 2 [file Data_Sheet_2.PDF]

Column Number of each percept/sensor in the MPL Dataset

Example 1 - column 5 in the dataset corresponds to the position percept for the Shoulder Abduction/Adduction joint

Example 2 - column 36 in the dataset corresponds to the temperature percept of the Index Finger (MCP) Flexion/Extension joint

Column Number	Joint	Percept
1	Shoulder Flexion/Extension	Position
2	Shoulder Flexion/Extension	Velocity
3	Shoulder Flexion/Extension	Load
4	Shoulder Flexion/Extension	Temperature
5	Shoulder Abduction/Adduction	Position
6	Shoulder Abduction/Adduction	Velocity
7	Shoulder Abduction/Adduction	Load
8	Shoulder Abduction/Adduction	Temperature
9	Humeral Rotation	Position
10	Humeral Rotation	Velocity
11	Humeral Rotation	Load
12	Humeral Rotation	Temperature
13	Elbow Flexion/Extension	Position
14	Elbow Flexion/Extension	Velocity
15	Elbow Flexion/Extension	Load
16	Elbow Flexion/Extension	Temperature
17	Wrist Rotation	Position
18	Wrist Rotation	Velocity
19	Wrist Rotation	Load
20	Wrist Rotation	Temperature
21	Wrist Abduction/Adduction	Position
22	Wrist Abduction/Adduction	Velocity
23	Wrist Abduction/Adduction	Load
24	Wrist Abduction/Adduction	Temperature
25	Wrist Flexion/Extension	Position
26	Wrist Flexion/Extension	Velocity
27	Wrist Flexion/Extension	Load
28	Wrist Flexion/Extension	Temperature
29	Index Finger Abduction/Adduction	Position
30	Index Finger Abduction/Adduction	Velocity
31	Index Finger Abduction/Adduction	Load
32	Index Finger Abduction/Adduction	Temperature
33	Index Finger (MCP) Flexion/Extension	Position
34	Index Finger (MCP) Flexion/Extension	Velocity
35	Index Finger (MCP) Flexion/Extension	Load
36	Index Finger (MCP) Flexion/Extension	Temperature
37	Index Finger (PIP) Flexion/Extension	Position
38	Index Finger (PIP) Flexion/Extension	Velocity
39	Index Finger (PIP) Flexion/Extension	Load

40	Index Finger (PIP) Flexion/Extension	Temperature
41	Index Finger (DIP) Flexion/Extension	Position
42	Index Finger (DIP) Flexion/Extension	Velocity
43	Index Finger (DIP) Flexion/Extension	Load
44	Index Finger (DIP) Flexion/Extension	Temperature
45	Middle Finger Abduction/Adduction	Position
46	Middle Finger Abduction/Adduction	Velocity
47	Middle Finger Abduction/Adduction	Load
48	Middle Finger Abduction/Adduction	Temperature
49	Middle Finger (MCP) Flexion/Extension	Position
50	Middle Finger (MCP) Flexion/Extension	Velocity
51	Middle Finger (MCP) Flexion/Extension	Load
52	Middle Finger (MCP) Flexion/Extension	Temperature
53	Middle Finger (PIP) Flexion/Extension	Position
54	Middle Finger (PIP) Flexion/Extension	Velocity
55	Middle Finger (PIP) Flexion/Extension	Load
56	Middle Finger (PIP) Flexion/Extension	Temperature
57	Middle Finger (DIP) Flexion/Extension	Position
58	Middle Finger (DIP) Flexion/Extension	Velocity
59	Middle Finger (DIP) Flexion/Extension	Load
60	Middle Finger (DIP) Flexion/Extension	Temperature
61	Ring Finger Abduction/Adduction	Position
62	Ring Finger Abduction/Adduction	Velocity
63	Ring Finger Abduction/Adduction	Load
64	Ring Finger Abduction/Adduction	Temperature
65	Ring Finger (MCP) Flexion/Extension	Position
66	Ring Finger (MCP) Flexion/Extension	Velocity
67	Ring Finger (MCP) Flexion/Extension	Load
68	Ring Finger (MCP) Flexion/Extension	Temperature
69	Ring Finger (PIP) Flexion/Extension	Position
70	Ring Finger (PIP) Flexion/Extension	Velocity
71	Ring Finger (PIP) Flexion/Extension	Load
72	Ring Finger (PIP) Flexion/Extension	Temperature
73	Ring Finger (DIP) Flexion/Extension	Position
74	Ring Finger (DIP) Flexion/Extension	Velocity
75	Ring Finger (DIP) Flexion/Extension	Load
76	Ring Finger (DIP) Flexion/Extension	Temperature
77	Little Finger Abduction/Adduction	Position
78	Little Finger Abduction/Adduction	Velocity
79	Little Finger Abduction/Adduction	Load
80	Little Finger Abduction/Adduction	Temperature
81	Little Finger (MCP) Flexion/Extension	Position
82	Little Finger (MCP) Flexion/Extension	Velocity
83	Little Finger (MCP) Flexion/Extension	Load
84	Little Finger (MCP) Flexion/Extension	Temperature
85	Little Finger (PIP) Flexion/Extension	Position
86	Little Finger (PIP) Flexion/Extension	Velocity

87	Little Finger (PIP) Flexion/Extension	Load
88	Little Finger (PIP) Flexion/Extension	Temperature
89	Little Finger (DIP) Flexion/Extension	Position
90	Little Finger (DIP) Flexion/Extension	Velocity
91	Little Finger (DIP) Flexion/Extension	Load
92	Little Finger (DIP) Flexion/Extension	Temperature
93	Thumb (CMC) Abduction/Adduction	Position
94	Thumb (CMC) Abduction/Adduction	Velocity
95	Thumb (CMC) Abduction/Adduction	Load
96	Thumb (CMC) Abduction/Adduction	Temperature
97	Thumb (CMC) Flexion/Extension	Position
98	Thumb (CMC) Flexion/Extension	Velocity
99	Thumb (CMC) Flexion/Extension	Load
100	Thumb (CMC) Flexion/Extension	Temperature
101	Thumb (MCP) Flexion/Extension	Position
102	Thumb (MCP) Flexion/Extension	Velocity
103	Thumb (MCP) Flexion/Extension	Load
104	Thumb (MCP) Flexion/Extension	Temperature
105	Thumb (IP) Flexion/Extension	Position
106	Thumb (IP) Flexion/Extension	Velocity
107	Thumb (IP) Flexion/Extension	Load
108	Thumb (IP) Flexion/Extension	Temperature

NOTES:
MCP - metacarpophalangal joint. This is the joint of a finger closest to the palm
PIP - proximal interphalangeal joint. This is the middle joint of a finger
DIP - distal interphalangeal joint. This is the joint of the finger furthest from the palm
CMC - carpometacarpal joint. This is the joint of the thumb closest to the palm
IP - interphalangeal joint. This is the joint of the thumb furthest from the palm

The units for each type of percept are as follows:
Position (rads)
Velocity (rads/s)
Load/Torque (Nm)
Temperature (degC)
